# A rare case of drug‐induced bradycardia associated with the just low dose use of methylprednisolone in a child with COVID‐19

**DOI:** 10.1002/jmv.27525

**Published:** 2021-12-20

**Authors:** Ping Jiang, Shuang‐hua Wu, Ting Zhu, Yuan Guo, Yi He

**Affiliations:** ^1^ Department of Cardiology Zhuzhou Central Hospital (Zhuzhou Hospital Affiliated to Xiangya School of Medicine) Zhuzhou China; ^2^ Critical Care Medicine, Zhuzhou Central Hospital (Zhuzhou Hospital Affiliated to Xiangya School of Medicine) Zhuzhou China; ^3^ Department of Hepatobiliary Surgery Zhuzhou Central Hospital (Zhuzhou Hospital Affiliated to Xiangya School of Medicine) Zhuzhou China

**Keywords:** coronavirus, infection, inflammation

## Abstract

Methylprednisolone (MP) is usually used to reduce inflammation reaction and tissue damage, which may have a beneficial treatment effect on coronavirus disease 2019 (COVID‐19). However, we present the case of a child who manifests significant bradycardia with the use of just low dose MP on the premise of the long‐term use of arbidol. Arbidol can affect the activity of CYP3A4, which is also a key metabolic enzyme of MP by competitive inhibition, and which is easy to aggravate the side effects of MP. Therefore, more attention should be paid to bradycardia occurrence in the patient with COVID‐19 when MP is considered in COVID‐19.

## Highlights

- The joint use of low dose MP and arbidol can also induce bradycardia in COVID‐19.
- The mechanism of the MP‐induced bradycardia need to be demonstrated in more detail.

## 1 INTRODUCTION

The severe acute respiratory syndrome coronavirus 2 (SARS‐COV‐2) infection has caused an outbreak of novel COVID‐19 since late 2019 and has rapidly become the greatest direct threat to worldwide health security. However, to date, no specific treatment is demonstrated to control and eradicate SARS‐COV‐2 significantly,^1^ therefore, a majority of treatment strategies for patients with COVID‐19 mainly depend on previous experience with the severe acute respiratory syndrome in 2003.^2^ Corticosteroids can be a benefit for the moderate‐to‐severe individual with COVID‐19 via suppressing severe systemic inflammation.^3^ We present the case that the just low dose use of methylprednisolone (MP) in the child with COVID‐19 is involved in sinus bradycardia, which is reported for the first time to escalate vigilance for clinicians in case of adverse events.

### 1.1 Case presentation

**Figure 1:**
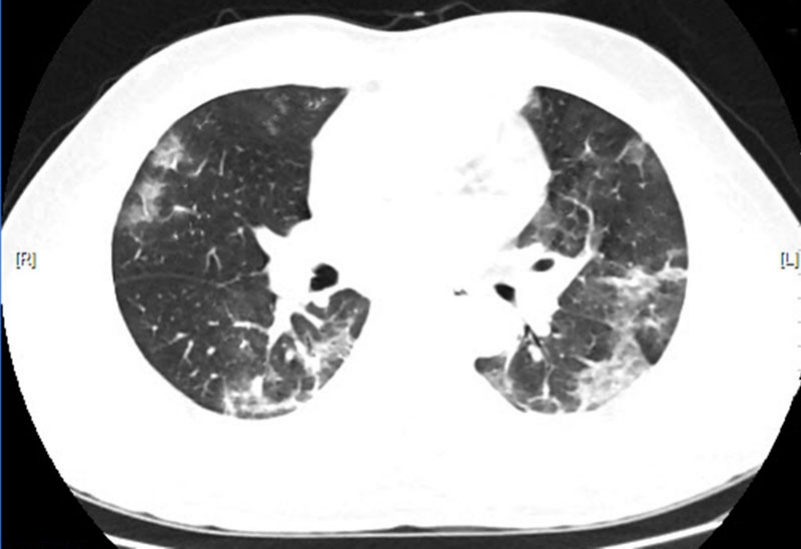
Thoracic computed tomography

**Figure 2:**
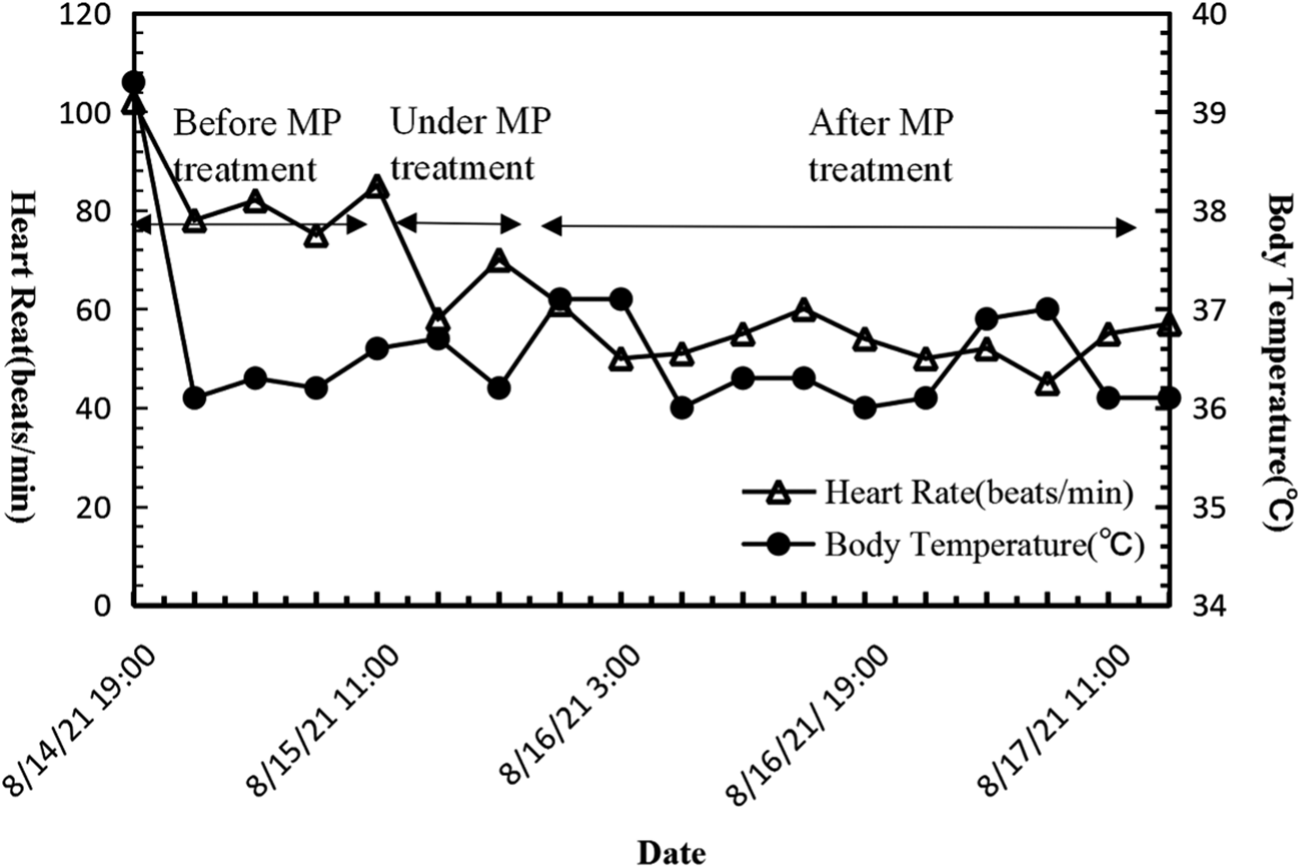
The change of heart rate and body temperature before, under, and after methylprednisolone (MP) treatment, respectively (arrows)

A 12‐year‐old previously healthy male had close contact with his elder sister who had been diagnosed COVID‐19 on July 23, 2021, he was brought to the hospital for cough and sore throat on August 7, 2021. His weight was 68 kg at admission. Both nasopharyngeal and oropharyngeal swabs for SARS‐CoV‐2 were tested positive by a reverse transcriptase‐polymerase chain reaction in admission. The level of C‐reactive protein (CRP), erythrocyte sedimentation rate (ESR), and procalcitonin (PCT) were normal in admission. Thoracic computerized tomography scans indicate that patchy ground‐glass opacities and high‐density plot areas were scattered in both lower lobes (Figure 1), accordingly, a typical COVID‐19 was diagnosed. Therefore, arbidol with 100 mg three times a day and interferons gamma were received for antiviral and immune‐modulatory treatment on August 9. However, he started to have a low fever up to 38°C 1 day later and developed a sustained high fever(over 39.0°C) with lower pulse oxygen saturation (about 93%) at his fingertip on August 14. Then, the clinical laboratory tests indicated CRP level of 200 ng/L (normal < 10 ng/L), ESR level of 100 mm/1 h (normal < 15 mm/1 h) and serum potassium level of 3.6 mmol/L (3.5 mmol/L < normal < 5.5 mmol/L). Nevertheless, the repeated laboratory tests at PCT and serum cardiac troponin T (cTnT) were always normal. The repeated transthoracic echocardiography (TTE) test was also normal and there was no abnormality in the electrocardiogram except only sinus bradycardia. In view of the acute exacerbation of the condition, the intravenous drip MP with 20 mg twice a day (∼0.58 mg/kg/day) was used for treatment to suppress severe systemic inflammation and alleviate the damage of the lung on August 15, however, the heart rate was reduced to 50–60 beats/min, when starting dose of 20 mg is used, then gradually returned to normal after running out of MP with 20 mg for 2 h. Although the patient remains asymptomatic and rectal temperature dropped to normal with the treatment of the second MP with 20 mg on the same day, the child's heart rate was found to seriously decline down to 40–60 beats/min at rest again (Figure 2), but both the level of cTnT and repeated TTE test were normal, fortunately, sinus bradycardia restored after MP was discontinued over a 1‐week period, and the patient was cured without comorbidities in accordance with clinical cure standard after 1 month.

## 2 DISCUSSION

As is known, although viral myocarditis may induce a variety of slow cardiac arrhythmia as a result of a direct or indirect myocardial injury caused by SARS‐CoV‐2, the diagnosis proof of suspected myocarditis is ruled out on account of the several negative results of myocardial injury marker and echocardiograph according to the diagnostic criteria for clinically suspected myocarditis.^4^ As is reported, no serious adverse effect such as bradycardia was found in arbidol treatment so far,^5^, ^6^ but in fact, a previous study showed that a high dosage of corticosteroids could cause sinus bradycardia.^7^ However, in our case, the patient with COVID‐19 manifests sinus bradycardia with the use of just a low dosage MP of 0.58 mg/kg/day, whereas the heart rate came back to normal level upon cessation of MP and arbidol. According to the adverse drug reaction probability scale (Table 1),^8^ the total score is 13, we evaluated a high score of 10, demonstrating a stronger causal relationship between low dose MP and the adverse clinical event, which is reported for the first time.

**Table 1:** Adverse drug reaction probability scale

	Yes	No	Don't know	Score
Are there previous conclusive reports on this reaction?	+1	0	0	+1
Did the adverse event appear after the suspected drug was administered?	+2	−1	0	+2
Did the adverse reaction improve when the drug was discontinued or a specific antagonist was administered?	+1	0	0	+1
Did the adverse reaction reappear when the drug was readministered	+2	−1	0	+2
Are there alternative causes (other than the drug) that could on their own have caused the reaction?	−1	+2	0	+2
Did the reaction reappear when a placebo was given?	−1	+1	0	0
Was the drug detected in the blood (or other fluids) in concentrations known to be toxic?	+1	0	0	0
Was the reaction more severe when the dose was increased, or less severe when the dose was decreased?	+1	0	0	+1
Did the patient have a similar reaction to the same or similar drugs in any previous exposure?	+1	0	0	0
Was the adverse event confirmed by any objective evidence?	+1	0	0	+1
			Total score	+10

But, to date, the mechanism of low dose MP caused bradycardia is not quite clear and also needs to be discussed in detail. On the one hand, a previous animal study indicated that MP can decrease cardiovascular beta‐1‐receptor sensitivity,^9^ which in turn, can reduce the excitability, automaticity, and rhythmicity of sinoatrial node cells. On the other hand, CYP3A4 in microsomes from the liver was identified as a key metabolic enzyme of arbidol,^6^ and MP also plays the role as a substrate of CYP3A4. Thereby, MP combined with arbidol will have potential drug–drug interactions which can prolong and enhance its effects and lead to drug side effects.

As is well‐known, MP has similar physiological effects of aldosterone which can lead to water–sodium retention, as a result, water–sodium retention with an increase of the volume load and pressure load could cause reflex heart rate decrease by carotid sinus stimulation.

## 3 CONCLUSION

When MP is determined to reduce lung injury and systemic inflammation reaction on the premise of the use of arbidol, the drug interaction between MP and arbidol should be considered to avoid adverse drug events.

## CONFLICT OF INTERESTS

The authors declare that there are no conflict of interests.

## Data Availability

The data that supports the findings of this study are available in the manuscript.
